# Uptake, metabolism and toxicity of iron oxide nanoparticles in cultured microglia, astrocytes and neurons

**DOI:** 10.1186/2193-1801-4-S1-L32

**Published:** 2015-06-12

**Authors:** Charlotte Petters, Ralf Dringen

**Affiliations:** University of Bremen, Germany

**Keywords:** Toxicity, protection, nanoparticles

Iron oxide nanoparticles (IONPs) are frequently used for biomedical applications including magnetic resonance imaging, drug delivery or tumor treatment by hyperthermia. Since IONPs can reach the brain from periphery or are directly applied into the brain, the investigation of potential adverse effects of IONPs on properties and functions of the different types of brain cells is an important task. In order to directly compare different types of brain cells concerning the uptake and toxicity of IONPs, we synthesized fluorescent IONPs and characterized these BODIPY-labeled particles regarding their physicochemical properties. In the medium used for the cell studies, these particles had a hydrodynamic diameter of around 158 ± 28 nm and a negative surface charge with a zeta-potential of -10 ± 2 mV. Exposure of primary cultures of rat astrocytes, neurons and microglial cells revealed that among these cell types, microglial cells accumulated IONPs most rapidly. However, microglial cells were also most vulnerable towards acute IONP-induced stress while astrocytes and neurons were not acutely damaged by IONPs. In microglial cells, but not in astrocytes or neurons, IONPs were found to be localized in lysosomes and large amounts of reactive oxygen species (ROS) were observed after IONP-exposure. The IONP-induced toxicity in microglial cells was prevented by neutralizing lysosomes or by chelation of intracellular ferrous iron ions, suggesting that the toxic potential of IONPs in microglia involves rapid particle uptake, liberation of ferrous iron from the internalized IONPs in the acidic lysosomal compartment and iron-catalyzed ROS formation. These data suggest that also in brain IONPs may harm microglial cells and compromise microglial functions.

